# Deutsches Optisches Museum – edutainment for optics & photonics

**DOI:** 10.1016/j.zemedi.2025.12.002

**Published:** 2025-12-22

**Authors:** Timo Mappes

**Affiliations:** Deutsches Optisches Museum, c/o Abbe Zentrum Beutenberg, Hans-Knöll-Str. 1, 07745 Jena, Germany; Friedrich-Schiller-Universität Jena, Physikalisch-Astronomischen Fakultät, Max-Wien-Platz 1, 07743 Jena, Germany

**Keywords:** Museum, Optics, Photonics, Medical physics, Science Communications, STEM (Science, Technology, Engineering, and Mathematics), Education, Edutainment

## Abstract

Currently under construction in Jena, Germany, the Deutsches Optisches Museum (D.O.M.) represents a novel concept in science communication, merging the principles of modern science centers with the heritage of classical museums. Designed as an interactive edutainment hub, the D.O.M. integrates artistic, aesthetic, and scientific approaches to engage the public in optics, photonics, and medical physics. Visitors will be driven to learn through curiosity, visual fascination, and hands-on experimentation, all of which will be complemented by in-depth scientific explanations. Spanning more than 3100 m^2^ across four floors, the museum’s exhibition will be dedicated to microscopy, ophthalmic optics and spectacle lenses, and astronomy. Key highlights include the world’s largest historical glass archive; interactive relay-optics stations that allow visitors to “look through” historical instruments; and large-scale educational models such as a walk-in human eye. The concept unites education, research outreach, and design through collaboration with leading architects, artists, scientists, and industrial partners. Located in the historical and contemporary heart of optical innovation, D.O.M. aims to set a global benchmark for communicating science in the fields of optics and photonics. The museum fosters public understanding and appreciation of the physical principles of optics and photonics that shape both medical and industrial technologies.

## Lighthouse of science communication in optics and photonics

A role model for the successful cooperation between academia and industry can be found in the heart of Germany. The ongoing collaboration between academic and public research institutions and the industry itself is the key to the success of the optical industry in Jena. Although this form of exchange has existed since the 19^th^ century, there is the need to establish it as a template for technology transfer in Europe. To emphasis the importance of optics and photonics, we are creating the Deutsches Optisches Museum (D.O.M.), a science communication center focusing on optics in the life and natural sciences. We combine art and the aesthetics of light with a playful yet serious education. In doing so we are creating a novel type of museum.

From a global perspective, Jena is the best location for this type of institution. Like a string of pearls, there are global milestones of optics and photonics, that were and are taken in this small town since 1801. Johann Wolfgang von Goethe initiated the discovery of ultraviolet light here in 1801 [1], and Ernst Abbe described the image formation in the wide-field microscope in Jena in 1873 [2]. In 1912, the ZEISS Punktal lens, the first modern precision spectacle lens ever, was introduced here[3]. In 1935, Alexander Smakula filed the patent for anti-reflective coatings on optics [4], originally designed for military applications and subsequently adapted for use on spectacle lenses. Teams from Jena have won the most relevant innovation prize in Germany, the Deutsches Zukunftspreis, in 2007, 2013, 2020, and 2022. No other region in Germany has a higher innovation density than Jena. Thus, it is a fair statement that billions of people are currently benefiting from the inventions created at the global hotspot of optics and photonics: Jena. Thus, here is the ideal location for the leading museum on optics and photonics.

The Deutsches Optisches Museum is currently under construction, enabled by the most prominent public-private-partnership in Germany. While the Optisches Museum was created as a scientific exhibition in 1922, and it had been open to the public since 1965 [5]. However, by 2016, the museum was outdated in all aspects, so Michael Kaschke, the CEO of Carl Zeiss AG at the time, initiated to entirely redo this endormant museum. In 2017, as a result, the foundation Deutsches Optisches Museum was established by the Carl Zeiss AG, the Carl Zeiss Foundation, the Ernst Abbe Foundation, the city of Jena, and the Friedrich Schiller Universität Jena. In 2018, the founding director was appointed Professor of History of Physics with a focus on Science Communication at Friedrich Schiller University Jena. He has served as the museum's scientific and managing director since then. While industry, foundations, the town, and a renowned university have joined forces, construction of the building and the exhibition itself are significantly supported as publicly funded projects. Funding bodies include the European Union, the Federal Government of Germany, and the State of Thuringia. To date, the private partners have committed to donating more than €32 million to the Deutsches Optisches Museum project.

## Global benchmark for optics and photonics in museums

The foundation Deutsches Optisches Museum has initiated the concept for and is creating this highly interactive exhibition, which blends education and entertainment. The general public will have access to more than 3,100 square meters where they can joyfully learn about optics and photonics. The D.O.M. will leverage the interactive nature of science centers by providing solid yet popular explanations of science while showcasing genuine historical objects. Thus, the visitors will experience the holistic integration of three elements:

(1) Interactive stations where they can experience and grasp the laws and phenomena of optics;
(2) Presentation on the implementation of optical principles in historical technical devices and instruments, as well as their applications in everyday life and science; and
(3) learning about latest research topics in the novel format of a “Showcase of Optics Research”.

With about 150 interactive stations, visitors will experience and connect the physical phenomena of optics to their own lives. In a playful manner, visitors will learn as they walk through the exhibition. For example, guests can melt metal using the power of the sun. Elsewhere, they can see the effect of sunscreen on their own skin using a UV camera and a sunscreen donor. Each interactive station explains the technical implementation and application. Visitors will experience how optics and photonics solutions are continuously changing our world. D.O.M. offers visitors a unique experience by enabling them to use historic optical instruments. If the mechanics of a historic museum object are not manipulated, it can be activated for user experience. By storing the historic microscopes in a climate-controlled showcase, we make their optics accessible via a simple type of telescope that penetrates the showcase’s glass. We call this kind of telescopes “relay-optics”. Visitors will be able to peer into these telescopes and see a microscopic image created by antique optics. We have tested this type of relay-optics and optimized it for people wearing spectacle lenses. We will adopt the same principle for selected spectroscopes and telescopes within our museum. While visitors typically only look at historical items in an exhibition, at D.O.M., they will look through them. Thus we will offer a unique authentic experience that reaches beyond the digital space.

Visitors will begin their exploration of each topic with an impressive yet meaningful installation. On the floor dedicated to ophthalmology, spectacle lenses, and medical optics, there will be a large model of the human eye that visitors can walk inside. Our guests will experience adjusting the large optical apparatus of that eye themselves to see images of two selected objects appearing to scale on the model of the retina (Fig. 2).

**Fig. 1:**
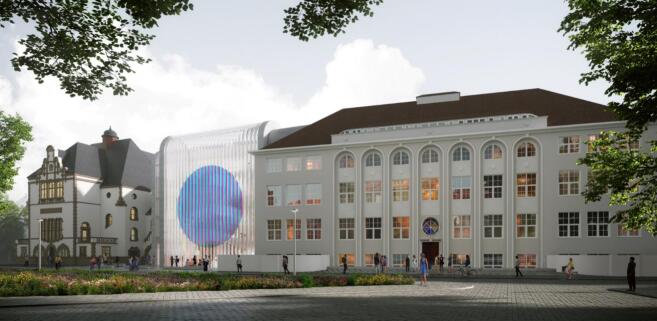
Iconic façade of Deutsches Optisches Museum in Jena: View towards the South of the listed building of the former State School of Opticians and the new building with its artistically designed façade by Studio Other Spaces (Olafur Eliasson and Sebastian Behmann) during daytime. Illustration by Luxigon for Studio Qwertz.

**Fig. 2:**
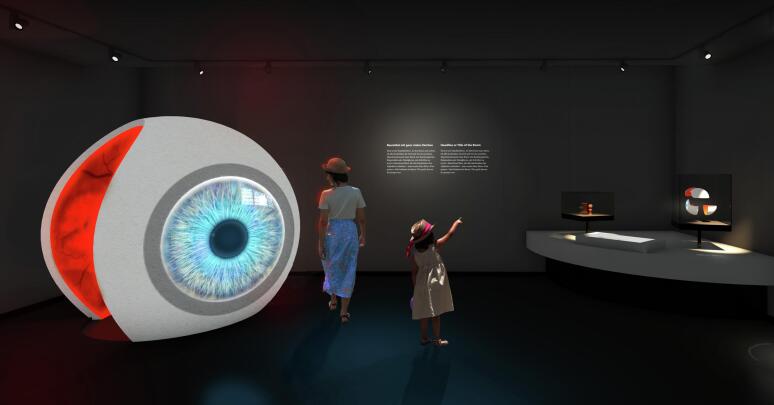
Rendering of the introduction room of the floor dedicated to ophthalmology, spectacle lenses and medical optics. Visitors will be able to use the large model of the human eye for imaging of selected objects. Illustration by Studio klv for D.O.M.

Additionally, we are developing a new type of science communication for recent optics research. Here, we aim to lower the barrier to engaging with cutting-edge research topics through a total of eight “Showcases of Optics Research”. Young scientists will present their latest research findings in a popular manner. D.O.M. will update these stations every six months. This ensures an inherent update of the exhibition, encouraging repeat visits.

**Fig. 3:**
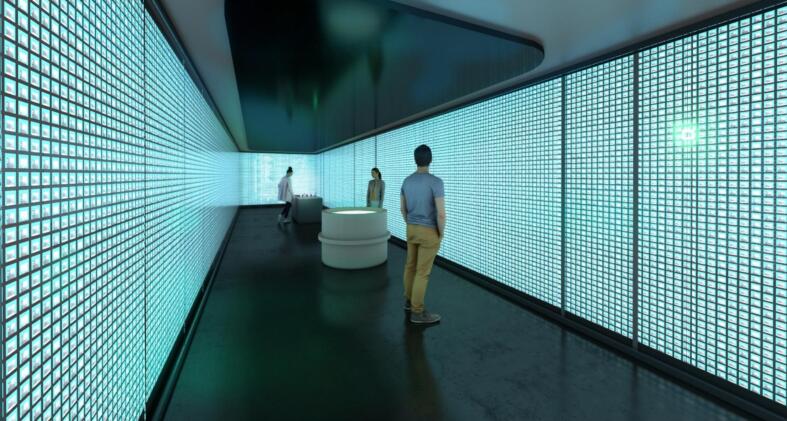
Take a walk to the world’s largest glass archive: More than 30,000 glass samples are on display in one immersive room as part of the world's largest glass archive. Visitors can select a lens from the production line on a screen, and the exact melts used in that lens will illuminate the wall. Additionally, artistic graphics will be displayed. Illustration by Studio klv for D.O.M.

We are creating the D.O.M. exhibition with our partner Studio klv from Berlin. The exhibition will comprise four floors of the former State School of Opticians’ listed building and a new building. One highlight will be the world's largest glass archive, which will be accessible in one room. Over 30,000 of our 140,000 glass samples from SCHOTT's production will be stored on the walls of this room and arranged artistically (Fig. 3).

In an experience that is unmatched in its authenticity, visitors will conduct the experiment that formed the basis of Johann Wolfgang von Goethe's color theory: Visitors to D.O.M. will create the interference spectrum themselves using the best-preserved prism that once belonged to Goethe. The prism is on loan from the museums of Klassik-Stiftung-Weimar. The same approach will be taken as with the historic microscopes: We are housing the genuine Goethe prism to maintain ideal conservation parameters. However, it will be positioned in a manner that allows visitors to use the prism viewing through it at a well-defined angle.

## Visibility of the D.O.M.

**Fig. 4:**
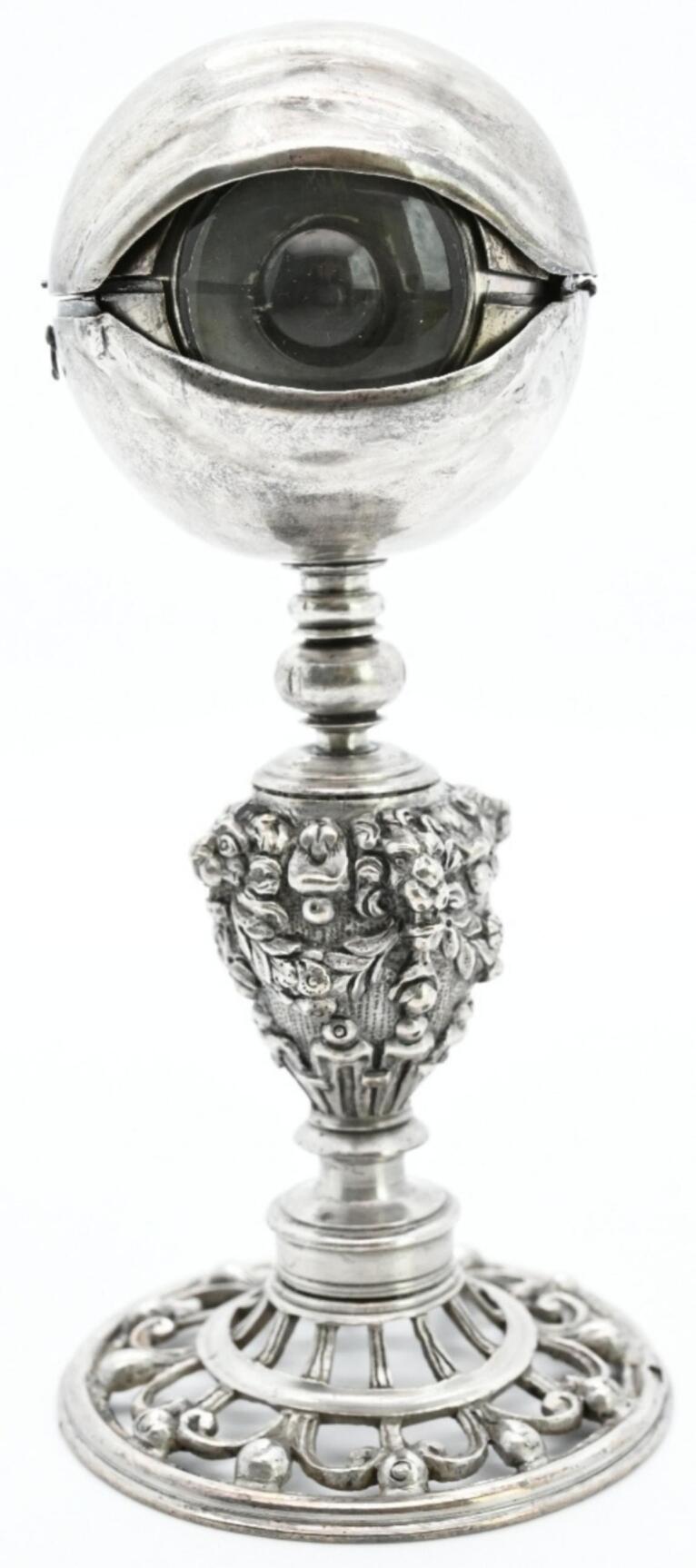
Silver model of a human eye, made around 1700 in France. Photo by Lisa Woop, D.O.M.

The D.O.M. will be open 358 days a year from 9 a.m. to 6 p.m. It will stay open until 8 p.m. on one weekday to accommodate evening visitors. The D.O.M. aims to become the premier tourist attraction and symbol of modern Thuringia. To this end, it is receiving significant support from tourism funding. Even long before its opening, the visibility of the D.O.M. collection is remarkable: In 2023, the D.O.M. lent items to the special exhibition “In Plain Sight” at the Wellcome Trust in London (October 20, 2022–February 12, 2023). Just one year later, in 2024, the D.O.M. lent the anatomical silver model of a human eye made in 1700 [6] to the special exhibition “Lumen” at the Paul J. Getty Museum in Los Angeles (September 10 – December 8, 2024) (Fig. 4). The Getty dedicated a postcard to this highlight.

## Architectural context

**Fig. 5:**
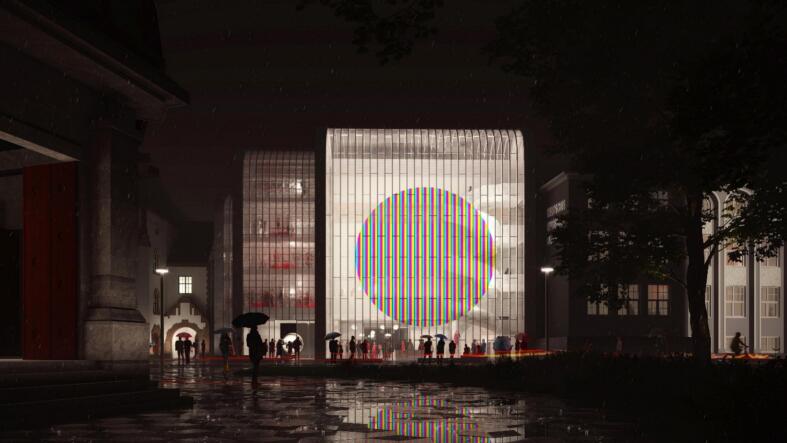
The colored glass stripes allow for a different perception of the building’s appearance. Illustration by Luxigon for Studio Qwertz.

Important spaces require attractive, compelling shells that symbolize their interiors and can serve as selfie backdrops or modern architectural icons. Following this idea, D.O.M.’s positioning as the holistic edutainment site for optics and photonics necessitates a grand architectural statement. Through Europe-wide bidding procedures, D.O.M. secured an exceptional team for planning and implementation:

Andreas Dopfer and his Berlin-based Studio Qwertz with its architects, who have repeatedly constructed museum buildings on UNESCO World Heritage sites, developed an iconic architecture consisting of four architectural bars with an exceptional façade. For this façade our team contracted Studio Other Spaces by Olafúr Elíasson and Sebastian Behmann. Olafúr Elíasson is an Icelandic-Danish artist known for his sculptures and large-scale installation. For example, he placed the sun in the Turbine Hall of the Tate Modern in London, UK, and with Sebastian Behmann he created the façade of the Harpa Reykjavik Concert Hall, which is the face of modern Reykjavik, Iceland. The new D.O.M. building is Studio Other Spaces' first architectural project in Germany and will become the icon of modern optics – and a new landmark. Specifically, the first architectural bar incorporates the 20-meter-high post-and-beam façade designed by Studio Other Spaces. Between the metal beam stripes, there is a 16-meter-diameter circle consisting of mouth-blown colored glass in magenta, yellow, and cyan (Fig. 5).

**Fig. 6:**
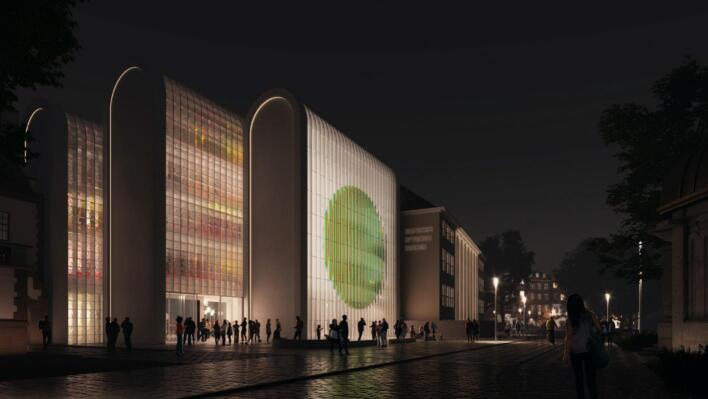
Artistic perspective at night: View of the new building from the East – the brightly lit atrium with its transparent and translucent façade invites to visit the museum or to linger in the café in the evening. Illustration by Luxigon for Studio Qwertz

The stained glass is arranged by the artists in a manner to create a stunning optical effect for passersby: the color of this circle changes dynamically (Fig. 1, Fig. 6).

We contracted Bartenbach GmbH from Austria to plan the lighting concept for the building and the exhibition. They have been creating the lighting concept for the Grand Egyptian Museum in Gizeh, Egypt.

The total project cost, including the renovation of the historic building and construction of the new entrance area and exhibition, amounts to €69 million. The new building provides barrier-free access to the entire complex and houses the museum café that will remain open beyond D.O.M.'s hours. The physics education department of Friedrich Schiller Universität Jena will use a dedicated room. All prospective physics teachers for the State of Thuringia will conduct their practical studies on optics here, ensuring early and sustainable STEM education. School classes will use this student lab in the afternoons. This part of the building contains a lounge intended for pupils to have their lunch during the day. In the evening, the lounge will be available for rent for small celebrations, offering a fantastic view of the D.O.M. atrium and its artistic façade.

For the architectural representation and staging of optics and photonics, there couldn't be a more suitable team of planners and artists worldwide. Since 2022, the D.O.M. has been a “premium project” of the National Urban Development Projects in Germany. In 2024, the D.O.M. began receiving funding from the Federal Government Commissioner for Culture and the Media as a “nationally important cultural institution”. That same year, the D.O.M. received the Polis Award 2024 for urban development. Construction is currently ongoing, and we intend to open to the public in 2028.

## CRediT authorship contribution statement

**Timo Mappes:** Writing – original draft, Funding acquisition, Conceptualization.

## Declaration of competing interest

The author declare that they have no known competing financial interests or personal relationships that could have appeared to influence the work reported in this paper.
